# Evaluation of the Potential of a Lectin Extracted from *Polygonum* *persicaria* L. as a Biorational Agent against *Sitophilus* *oryzae* L.

**DOI:** 10.3390/molecules27030793

**Published:** 2022-01-25

**Authors:** Mehdi Khoobdel, Vahid Rahimi, Asgar Ebadollahi, Patcharin Krutmuang

**Affiliations:** 1 Health Research Center, Lifestyle Institute, Baqiyatallah University of Medical Sciences, Tehran 1435916471, Iran; khoobdel@yahoo.com; 2 Department of Plant Sciences, Moghan College of Agriculture and Natural Resources, University of Mohaghegh Ardabili, Ardabil 5697194781, Iran; 3 Innovative Agriculture Research Center, Faculty of Agriculture, Chiang Mai University, Chiang Mai 50200, Thailand; 4 Department of Entomology and Plant Pathology, Faculty of Agriculture, Chiang Mai University, Chiang Mai 50200, Thailand

**Keywords:** carob extract, food attractant, lectin, *Polygonum persicaria*, *Sitophilus oryzae*

## Abstract

Rice weevil, *Sitophilus oryzae* L. (Coleoptera: Curculionidae), is one of the most destructive stored-product pests that is resistant to a wide range of chemical insecticides. In the present study, we investigated whether a lectin extracted from *Polygonum persicaria* L. (PPA) can be used as a biorational agent to control such insect pests. Along with the lethal digestive assay, the sub-lethal insecticidal activities of PPA, including the effects on digestive, detoxifying, and antioxidant enzyme activities, were evaluated against *S. oryzae* adults. The effect of feeding a diet containing PPA and carob extract as a food attractant on the mortality of *S. oryzae* adults was also investigated. Feeding on the diet containing PPA resulted in a significant mortality of *S. oryzae* adults with a LC_50_ (Lethal Concentration to kill 50% of insects) of 3.68% (*w*/*w*). The activity of digestive enzymes, including α-amylase, α-glucosidase, TAG-lipase, trypsin, chymotrypsin, elastase, and carboxy- and aminopeptidase, were decreased by the sub-lethal concentration of PPA. Detoxifying and antioxidant enzymes, including esterase, superoxide dismutase, catalase, glutathione-S-transferase, ascorbate peroxidase, glucose 6-phosphate dehydrogenase, and malondialdehyde, were activated in adults affected by PPA. These findings indicated that PPA, in addition to causing digestive disorders, leads to oxidative stress in *S. oryzae*. The presence of carob extract had no effect on the PPA-induced mortality of the insect. According to the results of the present study, PPA has promising insecticidal efficiency against *S. oryzae*. In addition, the usage of PPA with a food attractant carob extract in bait traps can be recommended as a new biorational formulation in *S*. *oryzae* management.

## 1. Introduction

A major concern for agriculture and the food industry is protecting crops from insects and pathogens. During storage, stored pests through the damage and contamination of grains cause main economic losses, impacting food security [1]. In general, chemical pesticides are used to control and manage pests, but these compounds may be harmful to humans, nontarget organisms, and the environment, as well as result in the development of insect resistance [2,3,4,5,6].

Rice weevil, *Sitophilus oryzae* L. (Coleoptera: Curculionidae), has been reported as one of the most destructive stored-product pests [7,8]. The adults invade the intact grains and feed on the seeds, and the larvae grow inside the grains. Thus, both the adults and larvae can reduce the quantity and quality of the stored grains [9]. Rice weevils have been reported to be resistant to many of the chemical pesticides and fumigants used for the protection of stored grains [5,10,11]. Chemical insecticides such as malathion, pyrimiphos methyl, phenytrothion, chlorpyrifos methyl, deltamethrin, permethrin, ciprofloxacin, diatomaceous particles, and spinosad as long-term preventers and protectors of the stored-product insects have been used. A major limitation on the use of chemical pesticides is insect resistance development, which has led to the cessation of some of these compounds as protectors of the pests in many countries. Today, the use of compounds such as malathion, methyl bromide, and dichlorvos, which were common pesticides in warehouses in the past, has been banned due to their severe side-effects [12]. The continuous use of phosphine as a prevalent fumigant to control the pest may induce resistance to this fumigant [13,14,15,16,17]. Stored-product insect resistance to phosphine has become a serious problem, and as there is no suitable alternative to this fumigant, it is very important to introduce and develop strategies to manage this problem. Thus, it is necessary to introduce insecticides that have no side-effects, and plant-based compounds can be a good strategy to manage this problem [18,19,20].

Plants use defense strategies against herbivorous insects, which include morphological traits and the synthesis of chemical substances, including secondary metabolites, as well as peptides and proteins [21]. One of these plant defense compounds is lectins or agglutinins [21,22,23], proteins that recognize specific carbohydrate structures and bind to them reversibly. Plant lectins have been reported to negatively affect biological and physiological traits of various insects, including the growth, survival, and oviposition of *Ephestia kuehniella* Zeller (Lepidoptera: Pyralidae), *Corcyra cephalonica* Stainton (Lepidoptera: Pyralidae), *Callosobruchus maculatus* Fabricius (Coleoptera: Chrysomelidae), and *Zabrotes subfasciatus* Bohemann (Coleoptera: Chrysomelidae) [24,25,26,27,28]. Lectins have been reported to bind to the insect peritrophic membrane, a chitinous matrix in the midgut with resistance to protease-based degradation [24,29,30,31,32]. These properties enable lectins to cause insect mortality and physiological and biological impairments [24]. It is proven that the lectins have toxicity and cause increased activity of detoxifying and antioxidant enzymes in insects [33]. 

The use of plant-based compounds such as lectins under storage conditions to control storage pests has been discussed. Using the method of “lure and kill” can be an interesting idea. Generally, compared to sprayable pesticides, poisonous baits are a safer method for pest control [34]. Mixing a food attractant with a toxin or insecticide has been used for many years in pest management [35]. One of the features of the mixtures is that they can be used effectively to control insects with lower residue on the plant or stored grains [35]. Currently, the toxic baits are used in the management of many pests, including cockroaches, ants, and house flies [34]. Attractants applied in the baits can be synthetic semiochemicals or crude extracts [36]. 

Identification of a wide range of attractants for stored-product insects such as *S*. *oryzae* and *Oryzaephilus surinamensis* L. (Coleoptera: Silvanidae) was performed for several years [37]. Cogan and Wakefield [38] showed that the crushed pods of carob trees are effective in attracting insects to pitfall traps. In addition, Collins et al. [37] found that the carob extract could be a food attractant for three pests of stored grain, including *Oryzaephilus surinamensis* (L.) (Coleoptera: Silvanidae), *Sitophilus granaries* L. (Coleoptera: Curculionidae), and *Cryptolestes ferrugineus* (Stephens) (Coleoptera: Laemophloeidae). There are several studies on the effect of food attractants on storage insects. Roberts et al. [39] and Wakefield and Morgan [40] reported that the carob extracts and volatiles are attractive for *O*. *surinammensis*.

The current study was conducted to investigate the insecticidal effectiveness of a lectin extracted from *Polygonum persicaria* L. (Polygonales: Polygonaceae) (PPA) against *S*. *oryzae*. Specifically, the toxicity mechanisms of the lectin, evaluating its effect on digestive, detoxifying, and antioxidant enzymes, along with the idea of whether it can be used as a poison bait for *S*. *oryzae* control, were considered in the present study.

## 2. Results

### 2.1. Purification of the Lectin

**Figure 1:**
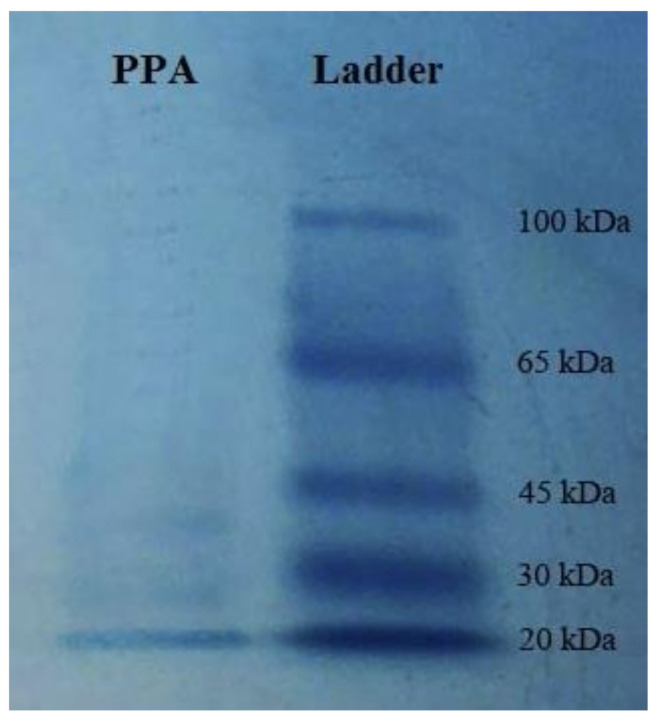
Molecular weight of the purified lectin from *Polygonum persicaria* (PPA) on SDS–polyacrylamide gel electrophoresis.

The purification process of the *P*. *persicaria* lectin indicated purified protein by the molecular weight of 20 kDa, approximately (Figure 1).

### 2.2. Bioassay

**Figure 2:**
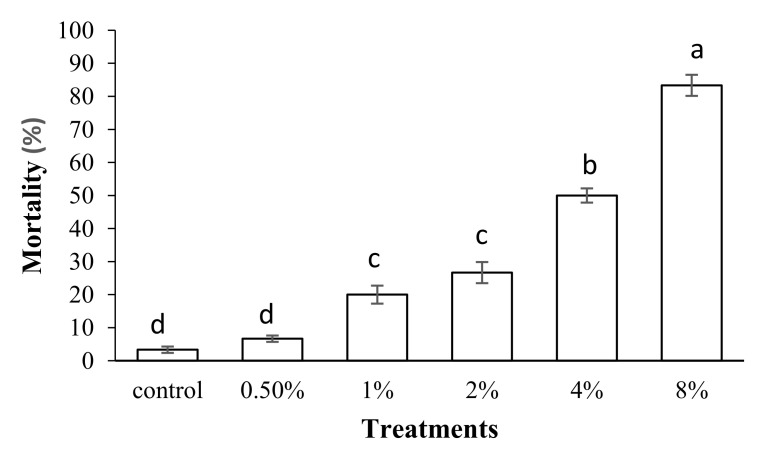
Effect of different concentrations of PPA on mortality percentage of Sitophilus oryzae adults. Means followed by different letters in each column are significantly different (*p* < 0.05, Tukey’s Test).

Our results revealed that feeding *S*. *oryzae* adults on a diet containing different concentrations of PPA caused significant mortality compared to the control (*p* < 0.05, df = 5) (Figure 2). The highest mortality was observed in adults fed on a diet containing 8% (*w*/*w*) PPA (Figure 2). The Probit analyses showed an estimated LC_50_ value of 3.68% (*w*/*w*) with a confidence limit of 2.65–5.78 and an LC_30_ value of 1.82% (*w*/*w*) with a confidence limit of 1.19–2.52 (Table 1). The results indicated that there is a positive and direct relationship between PPA concentrations and *S*. *oryzae* adult mortality (R^2^ = 0.95).

### 2.3. Effect on Digestive Enzymes of S. oryzae

Feeding on rice grains treated with a sub-lethal concentration of PPA affected the digestive enzymes’ activities in *S*. *oryzae* adults compared to the control. The activities of carbohydrases including α-amylase and α-glucosidase and also TAG-lipase were affected in *S*. *oryzae* adults fed on PPA. These digestive enzymes showed lower activities in *S*. *oryzae* adults treated with PPA (1.58, 0.33, and 0.26 U/mg protein for α-amylase, α-glucosidase, and TAG-lipase, respectively) compared to the control (4.28, 0.76, and 1.27 U/mg protein for α-amylase, α-glucosidase, and TAG-lipase, respectively) (*p* < 0.05, df = 7) (Table 2). In addition, the activities of specific proteases including trypsin (0.19 U/mg protein), chymotrypsin (0.17 U/mg protein), elastase (2.63 U/mg protein), and carboxy- (1.49 U/mg protein) and aminopeptidase (2.26 U/mg protein) statistically reduced in *S*. *oryzae* fed on rice grains treated with a sub-lethal dose of PPA, in comparison with the control (0.45, 0.46, 5.03, 4.88, and 5.11 U/mg protein for trypsin, chymotrypsin, elastase, and carboxy- and aminopeptidase, respectively) (*p* < 0.05, df = 7) (Table 2). 

### 2.4. Effect on Detoxifying and Antioxidant Enzymes of S. oryzae

**Figure 3:**
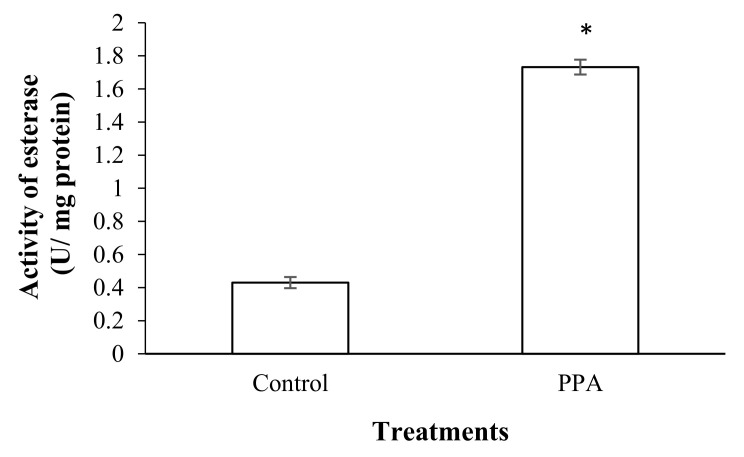
Activity of general esterase in control and PPA-treated adults of *Sitophilus oryzae*. Statistical differences have been marked by asterisks (*t*-test, *p* ≤ 0.05). * indicate significant deference.

**Figure 4:**
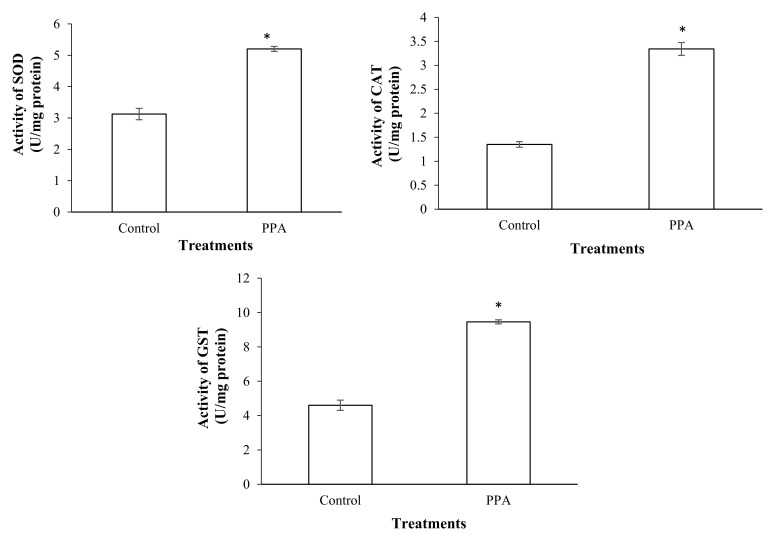
Activities of antioxidant enzymes in control and PPA-treated adults of *Sitophilus oryzae*: superoxidase dismutase (SOD); catalase (CAT); glutathione s-transferase (GST). Statistical differences have been marked by asterisks (*t*-test, *p* ≤ 0.05). * indicate significant deference.

**Figure 5:**
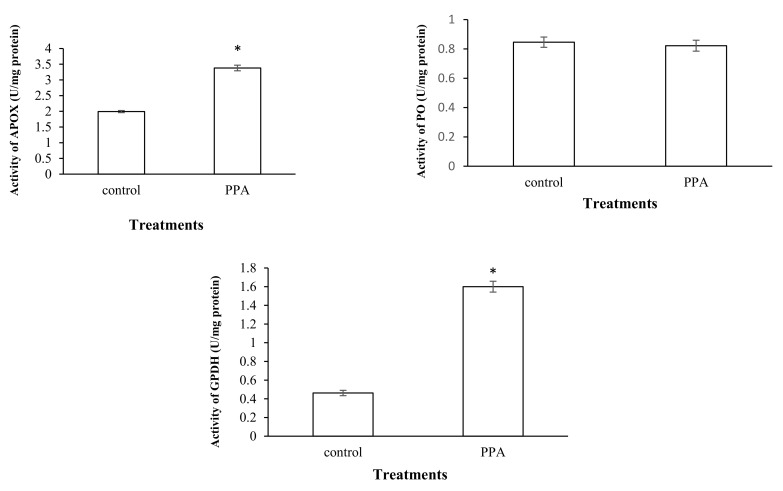
Activities of antioxidant enzymes in control and PPA-treated adults of *Sitophilus oryzae*: ascorbate peroxidase (APOX); peroxidase (PO); glucose 6-phosphate dehydrogenase (GPDH). Statistical differences have been marked by asterisks (*t*-test, *p* ≤ 0.05). * indicate significant deference.

**Figure 6:**
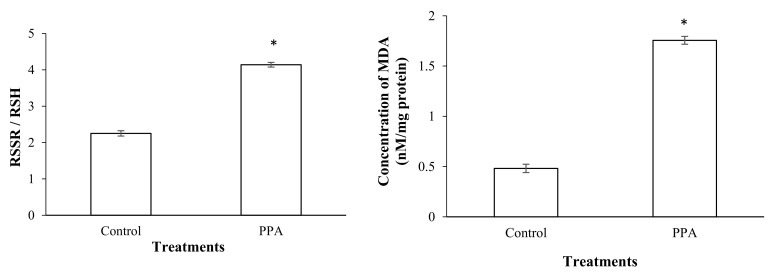
Ratio of oxidized to reduced thioles (RSSR/RSH) and MDA concentration in control and PPA-treated adults of *Sitophilus oryzae*. Statistical differences have been marked by asterisks (*t*-test, *p* ≤ 0.05). * indicate significant deference.

The results showed that a sub-lethal concentration (LC_30_) of PPA (1.82% (*w*/*w*)) caused a significant increase in the esterase activity of treated insects compared to the control (*p* < 0.05; df = 7) (Figure 3). The activities of antioxidant enzymes including superoxide oxidase (SOD) (*p* < 0.05; df = 5), catalase (CAT) (*p* < 0.05; df = 5), glutathione-S-transferase (GST) (*p* < 0.05; df = 7), ascorbate peroxidase (APOX) (*p* < 0.05; df = 5), and glucose 6-phosphate dehydrogenase (GPDH) (*p* < 0.05; df = 5) significantly increased in *S*. *oryzae* fed on PPA compared to the control (Figure 4 and Figure 5). However, the activity of peroxidase (PO) was not statistically changed in PPA-treated insects compared to the control (*p* > 0.05; df = 5) (Figure 5). The results showed that the RSSR/RSH ratio (*p* < 0.05; df = 5) and MDA (*p* < 0.05; df = 5) concentration in PPA-treated *S*. *oryzae* adults increased compared to the control (Figure 6).

### 2.5. Olfactory Assay

**Figure 7:**
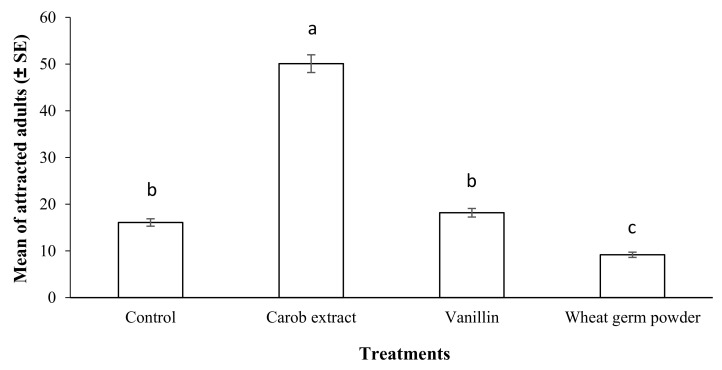
Mean number of adult Sitophilus oryzae attracted to each food attractant compared to control after a period of 12 h in a continuous-airflow multiple-choice olfactometer. Means followed by different letters in each column are significantly different (*p* < 0.05, Tukey’s Test).

The results of the olfactory assay by various food attractants are shown in Figure 7. The number of live *S*. *oryzae* adults in the tube containing a mixture of a rice grain, PPA, and carob extract powder (50.08) was significantly highest compared to other treatments and the control (*p* < 0.05; F = 246.37; df = 3) (Figure 7). These results suggested that carob extract powder is the best food attractant for *S*. *oryzae* adults due to the highest attractancy for the pest compared to others. 

### 2.6. Effect of a Mixture of PPA and Food Attractant on the Survival of S. oryzae

**Figure 8:**
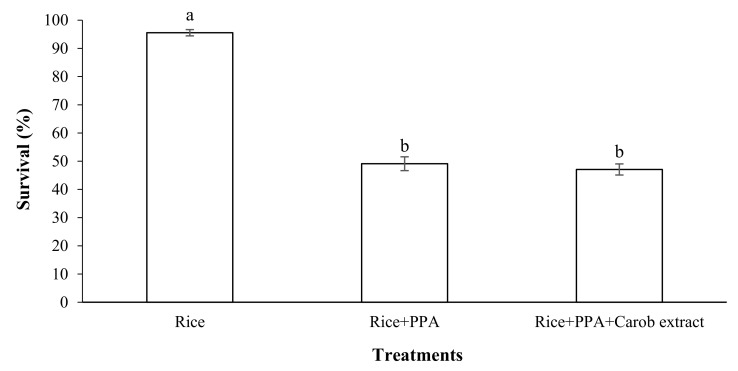
Effect of mixture of PPA and carob extract as food attractant on survival of Sitophilus oryzae adults (observation interval: 7 days). Means followed by different letters in each column are significantly different (*p* < 0.05, Tukey’s Test).

In this section, the survival of *S. oryzae* adults fed on a mixture of rice grains, LC_50_ PPA, and the attractant carob extract powder was estimated. The results showed that the adults of *S*. *oryzae* fed on diets containing a LC_50_ of PPA with carob extract have a similar survival than those without carob extract, but both are lower than that of the control (*p* < 0.05; F = 131.92; df = 3) (Figure 8).

## 3. Discussion

During recent decades, the use of biopesticides has increased because they are more environmentally friendly than chemical pesticides [26]. In recent years, an important development was made in the evaluation of the effect of plant lectins against insect pests [41]. Several reports have indicated that the plant lectins reduce performance indicators such as nutrition and reproduction in many insect orders such as Lepidoptera, Coleoptera, Diptera, and Hemiptera [42]. In the present study, we purified a lectin extracted from stems of *P*. *persicaria*. The results showed that this purified plant lectin is a protein with a molecular weight in the range of 200 kD. In previous studies, the effect of this purified lectin on cotton bollworm, *Helicoverpa armigera* Hübner (Lepidoptera: Noctuidae), was investigated by Rahimi et al. [43]. They reported that the lectin had an entomotoxic effect on *H*. *armigera*.

In the current study, our results indicated that feeding *S. oryzae* adults on a diet containing *P. persicaria* lectin (PPA) caused significant mortality compared to the control. Toxic and deleterious effects of PPA on *H. armigera* were reported by Rahimi et al. [43]. There are similar findings that have reported the mortality of several stored-product pests due to feeding on plant lectins. For example, Macedo et al. [27] reported that *Bauhinia monandra* Kurz (Fabales: Fabaceae) leaf lectin caused significant mortality in *Callosobruchus maculatus* and *Zabrotes subfasciatus*. It seems that the deleterious effects of plant lectins on insects are due to the toxicity and antinutritional effects of lectins [1,27,44,45,46,47].

The results showed that feeding *S*. *oryzae* on rice grains treated with PPA reduced the activity of digestive enzymes. Napoleao et al. [1] reported that a lectin from *Myracrodruon urundeuva* leaf had inhibitory effects on digestive enzymes α-amylase and proteases in adults of *Sitophilus zeamais*. Albuquerque et al. [47] showed that lectin extracted from *Microgramma vacciniifolia* rhizome reduced α-amylase and β-glucosidase activities in *S*. *zeamais*. Rahimi et al. [43] reported that PPA had inhibitory effects on digestive enzymes in *H*. *armigera* larvae. Overall, it seems that the reduction in digestive enzymes activities in the PPA-treated *S*. *oryzae* adults might be due to the (i) binding of the lectin to receptors in the insect gut epithelial cells or digestive enzymes that resulted in inhibition of the enzymes or reduction in their secretion; (ii) connection of the lectin to the insect gut epithelial cells leading to cytotoxicity and the subsequent decrease in the number of the cells that secrete the digestive enzymes [48,49]. According to several pieces of evidence that represent the cytotoxicity of plant lectins [33,43,50,51,52], the second hypothesis seems to be more plausible.

The results showed that LC_30_ PPA caused a significant increase in activity of esterase and antioxidant enzymes, as well as RSSR/RSH ratio and MDA concentration, in treated insects compared to the control. The enhancement of general esterase activity in PPA-treated *S*. *oryzae* adults indicated that the plant lectin has toxic effects and induces the detoxifying process in the treated insect. Similar results have shown that various plant extracts increase the activity of esterases in beetles [53,54,55]. Moreover, some studies have shown that oxidative stress induction would be an important component of the toxicity mechanisms for insecticides. Insecticides may induce oxidative stress that leads to the production of free radicals and changes in antioxidants’ activity [56,57,58]. In the current study, an increase in the level of the antioxidant enzymes in *S*. *oryzae* adults fed on PPA indicated that the plant lectin causes oxidative stress in the pest. Increasing SOD and CAT activities in *S*. *oryzae* adults fed on PPA showed oxidative stress in the pest. It seems that PPA uptake increases concentrations of radicals of superoxide and hydrogen peroxide, which induce activation of these enzymes [59,60,61]. GST is an important detoxifying and antioxidant enzyme that removes products of lipid peroxidation or hydroperoxides from cells [58]. The higher activity of GST in the PPA-treated adults of *S. oryzae* may be attributed to its role in the inactivation of lipid peroxidation products accumulated in the past due to destructive oxidative stress. APOX causes reductions in H_2_O_2_ and oxidized ascorbate [62]. Here, the APOX activity increases in PPA-treated insects compared to the control, indicating the role of PPA in the occurrence of the ascorbate oxidation process and accumulation of H_2_O_2_ in the treated insects. The GPDH activity increases in PPA-treated insects. In the glycolysis process, the reducing equivalents are transferred from the cytoplasmic pool of NADPH to the membrane of mitochondria by glycerol-3-phosphate shuttle. In the next step, the reducing equivalents are re-oxidized by transferring electrons across the membrane of mitochondria rather than NADH itself [63]. NADPH is the final reducing equivalent in the GSH-GSSG system to equilibrate chemical constitutions between dehydroascorbate and ascorbate. The RSSR/RSH (oxidized and reduced thiols) ratio and MDA concentration are indicators that show the occurrence of the radical oxidative process [58]. An increase in the ratio and MDA concentration indicates oxidative stress and lipid peroxidation. Malondealdehyde is a compound that is produced in the lipid peroxidation of polyunsaturated fatty acids in biological cells [58,64]. In the current study, the enhancement of RSSR/RSH ratio and MDA concentration in PPA-treated *S*. *oryzae* adults demonstrated that PPA induces the lipid peroxidation process after binding to epithelial cells of the insect midgut and, therefore, created cytotoxicity, a similar finding to that reported in *H*. *armigera* larvae fed on 1% PPA [33].

The results of the olfactory assay revealed that the carob extract powder is the best food attractant for *S*. *oryzae* adults compared to vanillin and wheat germ powders. The potential of carob extract as a food attractant for stored-product pests is well known [37,65,66]. Cogan and Wakefield [38] showed that the crushed pods of carob trees are effective in attracting insects to pitfall traps. In addition, Collins et al. [37] illustrated that carob extract could be a food attractant for three pests of stored grain including *Oryzaephilus surinamensis* (L.) (Coleoptera: Silvanidae), *Sitophilus granaries* L. (Coleoptera: Curculionidae), and *Cryptolestes ferrugineus* (Stephens) (Coleoptera: Laemophloeidae). Obeng-Ofori [67] studied the attractancy of different doses of carob extract to *Cryptolestes pusillus* (Schönherr) (Coleoptera: Laemophloeidae), *O. surinamensis*, and *Prostephanus truncatus* (Horn) (Coleoptera: Bostrichidae). Generally, studies have suggested that the carob extract could be a good food attractant for stored-product pests and so has the potential to be used in insecticide formulations such as bait traps.

Our results showed that the adults of *S*. *oryzae* fed on a diet containing LC_50_ PPA with carob extract have similar a survival than those without carob extract. The purpose of this experiment was to determine whether the presence of the food attractant in the diet could affect the PPA-induced mortality of *S*. *oryzae* or not. Given that there are (a diet containing PPA with and without carob extract) no significant differences between the two treatments, it can be concluded that the carob extract did not interfere with PPA-induced mortality.

## 4. Materials and Methods

### 4.1. Insect Rearing

Cultures of *S*. *oryzae* were kept on rice grains (as maintenance substrate) in plastic jars (20 × 30 cm) at 28 ± 1 °C, 50–60% RH, and 16:8 photoperiods [68]. The culture was maintained for ten generations from 2019 to 2020. This colony was as stock in our assessments.

### 4.2. Purification of the Lectin

Knotgrass, *P*. *persicaria* was collected from roadsides in Rasht, Guilan province, Iran. The lectin from *P. persicaria* agglutinin (PPA) was isolated from the stems of the plant. This lectin was purified by Sepharose 4B-galactose and DEAE-Cellulose fast flow columns. The 1,3-diamino propane was used for washing the columns. Before centrifugation at 6000 rpm for 20 min, the *P*. *persicaria* stems were incubated in 0.1 M Tris-HCl buffer (pH 7.1) at 4 °C for 72 h. The supernatant was filtered by filter paper (Whatman No.1) and precipitation of its proteins was performed by ammonium sulfate. The pellet was centrifuged (6000 rpm, 20 min) and then added to 0.1 M Tris-HCl buffer (pH 7.1). The suspension was dialyzed in the same buffer for 24 h [28]. Samples were added to the column of Sepharose 4B-galactose and were then washed by the 1,3-diamino propane buffer (20 mM). Fractions with the highest concentration of protein were collected, mixed, and then loaded to DEAECellulose fast flow [49]. The fractions were collected and then dialyzed in NaCl (0.5 M) and finally freeze-dried. Denaturing polyacrylamide gel electrophoresis (SDS–PAGE) was performed by the method of Laemmli [69] for the determination of molecular weight of the purified protein. The acrylamide concentration in the separating and stacking gels was 10% and 4%, respectively. Coomassie brilliant blue R-250 (Sigma-Aldrich, St. Louis, MI, USA) was used to stain the proteins on the polyacrylamide gel.

### 4.3. Bioassay

For the determination of lethal and sub-lethal concentrations of PPA, five concentrations of the freeze-dried powder (average diameter of 300 nm) of the lectin (0.5, 1, 2, 4, and 8% (*w*/*w*)) were prepared and mixed with rice grains (30 g) in a glass petri dish (100 × 20 mm) as the *S*. *oryzae* diet. Thirty *S*. *oryzae* adults (24-h-old, without considering the sex of the adults) were used per treatment at 28 ± 1 °C, 50–60% RH, and 16:8 photoperiod. The mortality of the insects was evaluated after seven days. Rice grains without PPA were used to feed insects in control groups. Per treatments include four replications (120 insects for each treatment). 

### 4.4. Effect on Digestive Enzymes Activities of S. oryzae

The estimated LC_30_ of PPA (1.82% (*w*/*w*)) was added to 30 g of rice grains. Thirty 24-h-old *S*. *oryzae* adults (without considering the sex of the adults) were randomly selected from the stock. They were caged on the diet for 72 h and used for enzyme assays. The rice grains without PPA were used as a control diet. Before centrifugation at 13,000 rpm for 15 min at 4 °C, the gut was dissected and homogenized with 0.15 M sodium phosphate buffer (pH 7.0). The supernatants were used as insect gut extract for assays of digestive enzymes’ activities [1].

### 4.4.1. α-Amylase

The assay was carried out following Bernfeld [70] using 1% soluble starch as the substrate. Briefly, 20 µL of the substrate was added to the reaction mixture containing 10 µL of insect gut extract and 50 µL of 20 mM Tris-HCl buffer (pH 5.5). The mixture was incubated at 30 °C for 30 min. Dinitrosalicylic acid (DNS: 100 µL) was added and then placed in boiling water for 10 min. Finally, the absorbance was read at 540 nm wavelength (Microplate reader Stat fax 3200, Awareness Technology Inc., Palm City, FL, USA).

### 4.4.2. α- and β-Glucosidases

The α- and β-glucosidase assays were carried out according to Silva and Terra [71], using 15 µL of gut extract mixed with 50 µL of universal buffer (0.02 M, pH 5.5) and 30 µL of the substrates p-nitrophenol-α-glucopyranoside (5 mM) and p-nitrophenol-β-glucopyranoside (5 mM) for α- and β-glucosidase, respectively. After 10 min, the absorbance was read at 405 nm wavelength (Microplate reader Stat fax 3200, Awareness Technology Inc., USA).

#### 4.4.3. Triacylglycerol (TAG)-Lipase

The assay was carried out following Tsujita et al. [72]. Briefly, the reaction mixture included 20 µL of gut extract, 40 µL of the substrate p-nitrophenyl butyrate (27 mM), and 100 µL of Tris-HCl buffer (20 mM, pH 5.5). The mixture was incubated at 30 °C for 1 min. The absorbance was read at 405 nm wavelength (Microplate reader Stat fax 3200, Awareness Technology Inc., USA) after that the reaction was stopped by adding 1 M NaOH.

#### 4.4.4. Serine Proteinases

Activities of trypsin, chymotrypsin, and elastase as serine proteases were measured by the method of Oppert et al. [73]. The reaction mixture included 5 µL of gut extract, 5 µL of substrates (BApNA (Na-a-benzoyl-L-arginine-p-nitroanilide) (1 mM) for trypsin, SAAPFpNA (N-succinyl-alanine-proline-phenylalanine-p-nitroanilide) (1 mM) for chymotrypsin, and SAAApNA (N-succinyl-alanine-alanine-alanine-p-nitroanilide) (1 mM) for elastase), and 35 µL of Tris-HCl buffer (20 mM, pH = 5.5). The mixture was incubated at 30 °C for 10 min and then the absorbance was read at 405 nm wavelength (Microplate reader Stat fax 3200, Awareness Technology Inc., USA).

#### 4.4.5. Exopeptidases

Activities of the two exopeptidases carboxy- and aminopeptidases were determined according to Oppert et al. [73]. Five microliters of the substrate hippuryl-l-phenylalanine for carboxy- and hippuryl-l-arginine for aminopeptidase were added to the mixture including 5 µL of gut extract and 35 µL of 20 mM Tris-HCl buffer (pH = 5.5). The reaction mixture was incubated at 30 °C for 10 min and then the absorbance was read at 405 nm wavelength (Microplate reader Stat fax 3200, Awareness Technology Inc., USA).

### 4.5. Effect on Detoxifying and Antioxidant Enzymes of S. oryzae

The sub-lethal concentration of PPA (1.82% (*w*/*w*)) was added to rice grains (30 g). Thirty *S*. *oryzae* adults (five replicates) were reared on the diet for 72 h and then used for detoxifying and antioxidant enzyme assays. The rice grains without PPA were used as a control diet. The insects were homogenized in 20 microliters of distilled water and centrifuged at 13,000 rpm for 10 min at 4 °C, and the supernatant was used as insect extract for evaluation of detoxifying and antioxidant enzymes activities.

#### 4.5.1. Determination of General Esterase Activity

The esterase activity was determined by Han et al. [74] using β-naphthyl acetate (5 mM) as the substrate. Briefly, 20 µL of the substrate was mixed with 50 µL of 1 mM fast blue RR salt and 10 µL of insect extract. The absorbance of the reaction mixture was read at 450 nm wavelength after five minutes (Microplate reader Stat fax 3200, Awareness Technology Inc., USA).

#### 4.5.2. Glutathione S-Transferase 

Habig et al.’s [75] method was used for the estimation of glutathione *S*-transferase (GST) with 20 µL of insect extract added to 135 µL of 10 mM phosphate buffer (pH = 7.0) containing 150 mM NaCl (PBS) and 50 µL of 20 mM 1-chloro-2,4-dinitrobenzene (CDNB) substrate. The absorbance of the reaction mixture was read at 340 nm wavelength (Microplate reader Stat fax 3200, Awareness Technology Inc., USA).

#### 4.5.3. Catalase

The catalase assay was carried out following Wang et al. [64] with 50 µL of insect extract mixed with 500 µL of 1% hydrogen peroxide. The mixture was incubated at 28 °C for 10 min and the absorbance was read as ΔA at 240 nm wavelength (Microplate reader Stat fax 3200, Awareness Technology Inc., USA). 

#### 4.5.4. Superoxide Dismutase

Estimation of superoxide dismutase (SOD) was carried out according to McCord and Fridovich [76]. Briefly, 50 µL of insect extract were added to 500 µL of SOD solution (70 μM NBT and 125 μM xanthine-diluted PBS, 100 µL of 5.87 U/mL xanthine oxidase solution, and 10 mg of bovine serum albumin). Then, the SOD and 2 mL of PBS were added to the mixture and incubated under darkness at 28 °C for 20 min. The absorbance of the reaction mixture was read as the ΔA at 560 nm wavelength (Microplate reader Stat fax 3200, Awareness Technology Inc., USA).

#### 4.5.5. Peroxidase

Peroxidase was evaluated according to Addy and Goodman [77] with 50 μL of insect extract, 250 μL of buffered pyrogallol (0.05 M pyrogallol in 0.1 M phosphate buffer pH 7.0), and 250 μL of 1% hydrogen peroxide. The absorbance of the mixture was read every 30 s for 2 min at 430 nm wavelength (Microplate reader Stat fax 3200, Awareness Technology Inc., USA). The activity was calculated by an extinction coefficient of oxidized pyrogallol (4.5 L∙M^−1^).

#### 4.5.6. Ascorbate Peroxidase

The activity of ascorbate peroxidase was performed according to Asada [62] with 50 μL of insect extract added to 150 μL of potassium phosphate buffer (67 mM, pH = 7.0), 200 μL of hydrogen peroxide (30 mM), and 70 μL of ascorbic acid (2.5 mM). The absorbance of the reaction mixture was read at 290 nm wavelength for 5 min (Microplate reader Stat fax 3200, Awareness Technology Inc., USA).

#### 4.5.7. Glucose 6-Phosphate Dehydrogenase (GPDH)

The GPDH was assayed according to Balinsky and Bernstein’s [78] method with a reaction mixture containing 100 μL of Tris-HCl (100 mM, pH = 8.2), 30 μL of MgCl_2_ (0.1 mM), and 50 μL of NADP (0.2 mM). In the next step, 50 μL of water, 50 μL of insect extract, and 100 μL of GPDH (6 mM) were added to the mixture, and the absorbance was read at 340 nm wavelength (Microplate reader Stat fax 3200, Awareness Technology Inc., USA).

#### 4.5.8. RSSR/RSH Ratio

In the first step, 1 M trichloroacetic and oxidized thiols (RSSR) were decomposed to form reduced thiols (RSH) for 20 min. Then, the RSSR was neutralized by NaOH. In the second step, 50 μL of insect extract were added to 500 μL of DNTB solution (0.1%) in PBS. The absorbance of the mixture was read at 535 nm wavelength (Microplate reader Stat fax 3200, Awareness Technology Inc., USA) after incubation for 10 min at 37 °C∙min. The concentration of RSSR was calculated as the difference between the final concentration of reduced thiols (RSH and RSSR) and the initial concentration of RSH in the sample as the ratio of RSSR to RSH [79].

#### 4.5.9. Malondialdehyde (MDA)

Based on the method of Bar-Or et al. [80], the reaction mixture with 50 μL of the insect extract and 100 μL of trichloroacetic acid (20%) was centrifuged at 15000 g for 10 min at 4 °C. The supernatant was added to 100 μL of TBA reagent (0.8%) and then incubated at 100 °C for 60 min. The absorbance of the mixture was read at 535 nm wavelength (Microplate reader Stat fax 3200, Awareness Technology Inc., USA). The MDA concentration was reported as the amount of MDA produced per mg of protein using a molar extinction coefficient of 1.56 × 105 M^−1^·cm^−1^.

#### 4.5.10. Protein Assay

The protein content of each sample (samples related to digestive, detoxifying, and antioxidant enzymes assay) was measured by the method of Lowry et al. [81] (recommended by Ziest Chem. Co., Tehran-Iran, Tehran, Iran).

### 4.6. Olfactometry

The olfactory assay was carried out to determine the better food attractant for *S*. *oryzae* adults. A four-way-choice olfactometer was used for the experiment. Tubes (25 cm × 5 cm diameter) of the olfactometer were filled with a mixture of rice grains and food attractants carob, vanillin, and wheat germ, separately. These tubes contained rice grains (25 g) mixed with 1.25 g of carob extract powder (Sigma-Aldrich) and PPA (LC_50_) (tube 1); rice grains (25 g) mixed with 1.25 g of vanillin powder (Sigma-Aldrich) and PPA (LC_50_) (tube 2) and rice grains (25 g) mixed with 1.25 g of wheat germ powder (Sigma-Aldrich) and PPA (LC_50_) (tube 3). The fourth tube contained rice grains (25 g) and PPA (LC_50_) as the control. Continuous airflow (at the rate of 250 cm^3^ per minute) was passed through the tubes by fans (D-Net, I.R. Iran) installed at the end of each tube. One hundred *S. oryzae* adults (24 h old) were starved for four days and released to the central arena of the olfactometer. After 12 h, the number of *S*. *oryzae* adults in the tubes was recorded [82]. The experiment was performed in 12 replicates. Between replicates, the tubes were cleaned completely and the bait positions in the tubes were switched. 

### 4.7. Effect of a Mixture of PPA and Food Attractant on S. oryzae Mortality

The experiment was carried out in a petri dish containing a mixture of rice grains (25 g), LC_50_ PPA, and the best food attractant (1.25 g) proven in the previous section. Thirty *S. oryzae* adults (24-h-old) were fed on the mixture and their mortality was evaluated after 7 days. Controls included rice grains without attractant and PPA (positive control), and the mixture of rice grains and PPA (LC_50_) without attractant (negative control). The test was performed with five replicates. 

### 4.8. Statistical Analyses

The concentration–mortality data were subjected to Probit analyses by POLO-Plus (LeOra Software, Berkeley, CA, USA) to calculate lethal and sub-lethal concentrations. All other data were compared by one-way analysis of variance (ANOVA) followed by the post hoc Tukey’s test (for tests related to olfactometry and the effect of a mixture of PPA and food attractant on *S*. *oryzae* mortality) and t-test (tests related to digestive, detoxifying, and antioxidant enzymes assay) at a 5% probability level using SAS 9.3 software [83].

## 5. Conclusions

In conclusion, due to the detrimental effects of chemical insecticides on human health and the environment, as well as the appearance and development of insect resistance to them, several studies focused on alternative methods for the control of pests. Currently, using phosphine is a prevalent method to control stored-product pests, especially rice weevil, *Sitophilus oryzae*. Although this compound is common for pest control in stores, there are many resistance reports to the phosphine in *S*. *oryzae*. Based on the mentioned points, we must focus on safe methods for pest control. It has been proven that plant lectins as eco-friendly and natural agents have deleterious effects on insects due to their toxicity and antinutritional properties. In the present study, we found that the lectin extracted from *P. persicaria* caused mortality in *S*. *oryzae* adults. This plant lectin has anti-nutritional effects and toxicity on *S*. *oryzae* because it caused oxidative stress in the pest. Therefore, it seems that this plant protein can have the potential to be used as a safe insecticide against rice weevils. One point is how this compound can be used in stores for pest control. It may be a good idea to use lectin as a toxic substance in bait traps. Thus, we have to add other components such as attractants to the formulation. It was found that carob extract is the best compound to attract *S*. *oryzae* adults compared to others. Therefore, the food attractant mixed with the PPA can be used in bait traps. Eventually, we need more comprehensive studies on the application of the toxic plant protein in *S*. *oryzae* controls. For example, field studies such as the evaluation of PPA toxicity on *S*. *oryzae* and its potential application in bait formulation under in-store conditions should be conducted. Additional studies on the effect of this compound on insect reproduction and the probable emergence of pest resistance along with its structure and effects on food grains should also be considered.

## Figures and Tables

**Table 1:** Results of PPA digestive toxicity against the adults of *Sitophilus oryzae*.

N	LC_30_ with 95% Confidence Limit (%)	LC_50_ with 95% Confidence Limit (%)	Slope ± SE	χ^2^ (df = 3)	R^2^
120	1.82 (1.19–2.52)	3.68 (2.65–5.78)	1.717 ± 0.149	5.28	0.95

N = Number of insects in bioassay.

**Table 2:** Digestive enzymes’ activities in *Sitophilus oryzae* adults treated with sub-lethal concentration of PPA (LC_30_ = 3.68%).

Enzymes	Treatments	
	Control	PPA
α-Amylase (U/mg protein)	4.28 ± 0.13 *	1.58 ± 0.10
α-Glucosidase (U/mg protein)	0.76 ± 0.02 *	0.33 ± 0.02
β-Glucosidase (U/mg protein)	0.71 ± 0.01	0.72 ± 0.01
TAG-Lipase (U/mg protein)	1.27 ± 0.06 *	0.26 ± 0.02
Trypsin (U/mg protein)	0.45 ± 0.01 *	0.19 ± 0.03
Chymotrypsin (U/mg protein)	0.46 ± 0.01 *	0.17 ± 0.01
Elastase (U/mg protein)	5.03 ± 0.06 *	2.63 ± 0.15
Carboxypeptidase (U/mg protein)	4.88 ± 0.19 *	1.49 ± 0.12
Aminopeptidase (U/mg protein)	5.11 ± 0.05 *	2.26 ± 0.27

* Means with asterisks in each row are significantly different according to independent Student’s *t*-test at 5%.

## Data Availability

The data that support the findings of this study are available upon request from the authors.

## Sample Availability

Samples of the compounds are available from the authors.
